# Eco positioning drives sustainable fashion consumption through process related strategies and brand familiarity

**DOI:** 10.1038/s41598-025-02233-2

**Published:** 2025-05-21

**Authors:** Wenze Jian, Ziqi Zhong

**Affiliations:** Department of Management, The London School of Economics and Political Science, London, WC2A 2AE UK

**Keywords:** Fashion marketing, Eco-positioning, Consumer perception, Sustainability, Mathematics and computing, Applied mathematics, Computational science, Computer science, Information technology, Pure mathematics, Scientific data, Software, Statistics

## Abstract

This study investigates how eco-positioning strategies influence consumers’ evaluations of fashion brands, their willingness to pay for eco-friendly fashion products, and their sustainable fashion consumption intentions. Based on the Theory of Planned Behavior and the Value-Belief-Norm Theory, this study constructs an integrated analysis framework. Data were collected through a structured online experiment, wherein participants completed three randomized experimental modules, each testing a distinct dependent variable. Within each module, participants were independently assigned to different eco-positioning stimuli. The results indicate that eco-positioning significantly affects brand evaluation and purchase intention, with process-related eco-positioning having a stronger effect. High brand familiarity enhances the effectiveness of eco-positioning strategies. Strong eco-positioning remarkably increases consumers’ willingness to pay, with perceived environmental sustainability playing an important mediating role. Additionally, sustainable fashion consumption intention under eco-positioning advertising is markedly higher than that under other advertising conditions, with environmental concern and fashion involvement acting as key moderating factors.

## Introduction

Over the years, the fashion industry has faced increasing pressure regarding environmental protection and sustainable development. As global environmental issues become more severe, consumer interest in sustainable fashion has steadily risen^1^. Being one of the main contributors to global pollution and resource consumption, the fashion industry is gradually transitioning towards more sustainable business models^2,3^. Against this backdrop, eco-positioning, which involves promoting a brand’s environmental sustainability efforts, has become a key strategy in fashion marketing^4,5^. Eco-positioning is a way for brands to communicate their environmental commitments and a vital means to attract environmentally conscious consumers. As consumers become more aware of environmental issues, they increasingly consider the environmental impact of products and the sustainable practices of brands when shopping^6,7^. Fashion brands should produce their products and demonstrate their commitment to the environment. Sun and Yoon (2022)^8^ and Kreczmańska-Gigol and Gigol (2022)^9^ indicated that consumers were willing to pay a premium for eco-friendly products. Moreover, consumers showed higher loyalty to brands that emphasize environmental and sustainability aspects in their marketing. Therefore, effectively implementing eco-positioning is crucial for fashion brands to stand out in a competitive market. Despite its growing importance in fashion marketing, the specific impacts of eco-positioning on consumer perceptions and behaviors remain under-researched^10,11^. Most studies have focused on factors influencing consumer attitudes toward sustainable fashion and the barriers to sustainable fashion consumption^12–16^. However, research on how different eco-positioning strategies specifically affect consumer evaluations of fashion brands and their sustainable fashion consumption intention is still limited^17^. Additionally, the role of brand familiarity as a key variable in moderating the impact of eco-positioning on consumer perceptions and behaviors has not been fully explored^18^. Brand familiarity may influence how consumers interpret and respond to eco-positioning information, thus significantly affecting marketing outcomes. Understanding the interaction between brand familiarity and eco-positioning strategies is pivotal for fashion brands to effectively communicate their environmental commitments and promote sustainable consumption^19,20^.

Previous studies primarily focused on the cognitive and psychological barriers consumers face when choosing sustainable fashion products. For example, Mandarić et al. (2022) noted that environmentally conscious consumers were often willing to support sustainable brands. However, high prices and lack of information remained major factors influencing their purchasing decisions^21^. Kripa et al. (2021) also found that consumers held positive attitudes toward green brands. However, they chose lower-priced products that met their needs during actual purchases, revealing the “attitude-behavior” gap in green consumption^22^. Additionally, research indicated that many consumers did not fully understand the deeper meaning of “sustainability” when purchasing sustainable fashion products, leading to misconceptions about brands’ eco-positioning. Pérez et al. (2022) highlighted that consumers’ perceptions of sustainable fashion were mainly focused on environmental protection, overlooking factors such as social responsibility and fair labor practices in the production process^23^.

Although eco-positioning, as a brand marketing strategy, has been widely applied across many industries, research on its specific impact on fashion marketing remains insufficient. Existing studies primarily focus on eco-friendly product design, green production processes, and brand image building. For example, Zhang et al. (2024) explored the impact of green advertising appeals on consumer purchase intention, emphasizing the moderating role of environmental concern in this process^24^. The study revealed that green advertising appeals demonstrated greater effectiveness in boosting purchase intention among consumers with high environmental concerns. This finding established a theoretical foundation for examining advertising content design in eco-positioning strategies. Additionally, Soomro et al. (2025) focused on the response mechanisms of consumers to green advertising appeals. They specifically analyzed the moderating effects of individuals’ sensitivity to normative influence and their need for uniqueness in the acceptance of green information^25^. This study further revealed that different consumers, when exposed to similar green advertising messages, exhibited varied behavioral responses due to differences in their sensitivity to social norms and individual traits. These findings offered vital empirical support for this study’s discussion on the effects of diverse eco-positioning strategies on consumer behavior. However, the application of eco-positioning strategies in fashion brands and their specific effects on consumer behavior have not been thoroughly explored. Particularly, the differential influence of product-related and process-related eco-positioning strategies on brand evaluation, willingness to pay, and sustainable consumption intentions remains understudied.

In the current literature, some scholars have examined consumers’ purchase intentions toward eco-friendly products. However, few studies have conducted comparative analyses of the specific impacts of different eco-positioning strategies, such as product-related versus process-related eco-positioning, on consumers. In particular, little research has explored how these strategies operate through psychological factors like brand evaluation, purchase intention, and willingness to pay. Additionally, the moderating effects of factors such as brand familiarity, environmental awareness, and fashion involvement on these relationships have rarely been delved into. Therefore, this study addresses this research gap by investigating how diverse eco-positioning strategies influence consumers’ evaluations of fashion brands, willingness to pay, and sustainable consumption intentions. Thus, it provides theoretical support and practical guidance for fashion brand marketing strategies.

In recent years, research on sustainable fashion marketing has focused on three aspects: (1) Kaner et al. (2022) studied the communication effectiveness of environmental demands; (2) Farzin et al. (2023) explored the psychological mechanism of premium payment; (3) Lee et al. (2023) studied the selection strategy of advertisement types. Moreover, Wang et al. (2022) revealed that eco-labels influenced purchase intention through the mediation of visual attention. Lobato-Calleros et al. (2022) found that product type in a circular economy model moderated consumer acceptance. However, most studies still exhibited limitations in uncovering moderating mechanisms, as exemplified by Nandkeolyar et al. (2023)’s omission of emotional pathways. Studies also showed constrained coverage of moderating variables, illustrated by Xu et al. (2024)’s failure to incorporate cultural differences. Despite significant progress, the existing literature has three main limitations. First, most studies treat eco-positioning as a unidimensional construct (Table 1), overlooking the differential impacts of product-related and process-related positioning. Second, only a few empirical studies consider the moderating role of brand familiarity, raising questions about the generalizability of these strategies. Third, research on advertising effects often focuses on single information types, lacking dynamic interaction analyses between environmental values and fashion involvement.

**Table 1:** Research progress and gaps in sustainable fashion marketing.

Author (year)	Research focus	Methodology	Main findings	Research gap	Moderator variables	Mediator variables
Kaner et al. (2022)^26^	The impact of green advertising on brand attitude	Questionnaire	Environmental demands enhance brand credibility.	Unclassified positioning types and lack of process dimension investigation	N/A	N/A
Farzin et al. (2023)^27^	Drivers of willingness to pay	Joint analysis	The acceptance rate of premium for eco-friendly materials has reached 23%.	Neglecting the moderating effect of brand familiarity	Perceived quality	Income level
Lee et al. (2023)^28^	Comparison of sustainable advertising types	Experimental method (3 × 2 design)	The effectiveness of third-party authentication advertising is better than that of self-proclaimed advertising.	Contextual analysis without considering environmental values	Brand trust	Advertising credibility
Wang et al. (2022)^29^	The effect of eco-labels on purchase intention	Eye movement test	Eco-labels significantly attracted visual attention and enhanced purchase intention	The mediating role of consumers’ knowledge level was not discussed.	Visual attention	Environmental protection knowledge level
Lobato-Calleros et al. (2022)^30^	Consumer acceptance under a circular economy model	Mixed method (questionnaire + interview)	Consumers’ acceptance of rental fashion was affected by product types and usage scenarios.	The moderating effect of brand reputation was not analyzed.	Perceived utility	Product type
Xu et al. (2024)^31^	The green marketing effect of social media	Longitudinal tracking data	User-generated content could promote sustainable consumption behavior better than brand content.	Lacked cross-regional verification of cultural differences	Social identification	Cultural values
Nandkeolyar et al. (2023)^32^	Fast fashion and sustainable brand competition strategy	Game theory model	Sustainable brands should strengthen environmental protection certification to maintain market share under price competition.	Mediating path without considering consumer sentiment	N/A	Market competition intensity
Jäger et al. (2020)^33^	The impact of virtual fitting technology on green consumption	Laboratory experiments	Virtual fitting indirectly promoted environmentally friendly behavior by reducing the return rate.	Boundary conditions for unverified technical acceptance	Return intention	Technical familiarity
Dobrenova et al. (2019)^34^	Emotional appeal in moral fashion advertising	Content analysis method	Emotional narration was easier to stimulate consumers’ empathy than rational appeal.	Did not distinguish between different emotional types (e.g. guilt vs. pride)	Emotional resonance	Personal values
Li et al. (2023)^35^	Trust mechanism of consumers on second-hand fashion platforms	Structural equation modeling	Platform transparency and seller evaluation system positively affected trust and purchase intention	The moderating role of economic incentives was not analyzed.	Platform trust	Price sensitivity
This study	The multi-dimensional impact mechanism of environmental positioning	Triple experimental design	The process positioning effect is stronger than the product positioning effect.	Filling in gaps in type comparison, boundary conditions, and interaction effects	Perceived environmental sustainability	Brand familiarity and environmental concern

In Table 1, this study systematically addresses three core gaps. (1) Through experimental deconstruction of product-related versus process-related positioning, it reveals the heterogeneous impact mechanisms of eco-positioning strategies; (2) By introducing brand familiarity as a key moderating variable, it establishes a theoretical link between “positioning type-brand equity-consumer response”; (3) By innovatively employing an interaction design of advertising type × values, it overcomes the single-path explanatory limitations of existing research. These multi-dimensional breakthroughs provide a new theoretical lens for sustainable fashion marketing.

This study employs a mixed-methods approach to investigate how different eco-positioning strategies impact three key consumer responses: brand evaluation, willingness to pay, and sustainable consumption intentions. The experimental design uses multiple measurement techniques, including Likert scales to assess brand attitudes and purchase intentions, and a slider scale to measure willingness to pay for eco-friendly products. The research examines both direct effects of eco-positioning and mechanistic pathways, specifically exploring how brand familiarity moderates consumer responses and how perceived environmental sustainability mediates willingness to pay. This comprehensive framework allows for detailed analysis of the psychological processes underlying consumer reactions to different eco-positioning strategies in the fashion domain, addressing significant gaps in the existing literature on sustainable marketing.

## Literature review

In recent years, environmental awareness among consumers has constantly improved, and the concept of sustainable development has also been widely disseminated. More and more research has concentrated on applying eco-positioning in fashion marketing and its impact on consumer attitudes and behaviors. Current studies extensively utilize theories such as the Theory of Planned Behavior (TPB), the Elaboration Likelihood Model (ELM), Signaling Theory, and Schema Theory^36^. Tiwari et al. (2024) argued that individual behavioral intentions were influenced by attitudes, subjective norms, and perceived behavioral control. This theory was widely applied in fashion marketing to explain consumers’ motivations for sustainable fashion consumption^37^. Cairns et al. (2022) indicated that consumers’ attitudes toward eco-friendly fashion products, perceptions of social norms, and a sense of control over their purchasing behavior all affected their purchase intentions. Eco-positioning acted as a signal that helped brands convey their environmental sustainability and high product quality to consumers, thus increasing their willingness to pay^38^. Louis and Lombart (2024) discovered the influence of brand environmental information on consumer purchase intentions. Their research suggested that communicating environmental commitments could remarkably enhance consumers’ purchase intentions^39^. Vo and Wu (2022) demonstrated that the effectiveness of persuasive communication depended on the level of cognitive elaboration consumers engaged in when processing information. The ELM divided information processing into central and peripheral routes: the former focused on the content and quality of information, while the latter considered external cues such as emotions and attractiveness^40^. In fashion marketing, Castelló-Martínez (2023) suggested that eco-positioning, as specific information about a brand’s environmental commitment, could trigger central route processing in consumers, thus enhancing brand evaluation and purchase intentions^41^. Muisyo et al. (2022) validated the application of eco-positioning in fashion brand marketing. They discovered that detailed and credible environmental information markedly improved consumer attitudes and purchase intentions^42^. Carrión-Bósquez et al. (2024) indicated that the detail and authenticity of ecological information were crucial factors influencing consumer attitudes^43^. Salnikova et al. (2022) believed that brands could enhance product quality and value perceptions by conveying environmental commitment signals^44^. Taillie et al. (2024) explored the impact of eco-labels on consumers’ quality perceptions and willingness to pay. It showed that environmental certifications could effectively increase consumer trust and willingness to pay^45^. Kolović et al. (2023) confirmed the influence of brand environmental signals on consumer purchase decisions. They pointed out that the effectiveness of these signals relied on the credibility and consistency of the brand’s environmental commitments^46^. Qahri-Saremi and Montazemi (2023) emphasized how consumers’ prior knowledge and experience affected their perception and evaluation of new information^47^. Karaosman et al. (2020) noted that brand familiarity, as a form of prior knowledge, could impact consumers’ reactions to eco-positioning strategies. Schema theory was used in fashion marketing to explain how brand familiarity influenced the acceptance of a brand’s environmental commitments^48^. Watson et al. (2024) found that familiar brands were more likely to gain consumer trust and support when delivering environmental information^49^. Wang and Walker (2023) discussed the moderating role of brand familiarity in eco-marketing. They stated that consumers’ familiarity with a brand could affect their evaluation of its environmental measures and purchase intentions^50^.

In summary, domestic and international research on eco-positioning in the context of fashion marketing has made significant progress in understanding its impact on consumer perception and behavior. Studies have primarily focused on the mechanisms through which eco-positioning influences consumer attitudes and behavioral intentions. Moreover, they emphasize the application of theories such as the Signaling Theory, TPB, and ELM in explaining consumer sustainable consumption behaviors. TPB posits that consumers’ behavioral intentions are determined by three major factors: attitudes, subjective norms, and perceived behavioral control. In the context of eco-positioning strategies, consumers’ attitudes toward brands are often influenced by the brand’s ecological image. For example, brands that convey signals of environmental protection and social responsibility through eco-positioning can improve consumers’ attitudes toward the brand, thus enhancing their purchase intentions. The “subjective norms” in TPB can also influence consumers’ behavioral choices through social influences, particularly the growing environmental protection awareness. Perceived behavioral control is reflected in consumers’ self-efficacy in choosing eco-friendly products. Eco-positioning strategies, by enhancing the brand’s environmental image, can strengthen consumers’ sense of control over their eco-friendly purchasing behaviors. The ELM suggests that changes in consumer attitudes depend on their chosen information processing routes—central and peripheral. In the context of eco-positioning, consumers are highly concerned about environmental issues and possess strong environmental values. They may process eco-positioning information through the central route, carefully evaluating whether the brand aligns with their environmental standards. However, for consumers with lower environmental awareness, information may be processed through the peripheral route. They rely more on external signals (e.g., celebrity endorsements, eco-labels) rather than an in-depth understanding of the product’s specific environmental attributes. Therefore, eco-positioning strategies have varying effects across different consumer groups. Signaling Theory emphasizes that brands convey their intrinsic quality or commitment through externally visible signals, such as product ecological attributes and eco-certifications. In fashion marketing, eco-positioning strategies serve as “signals,” helping brands communicate their environmental commitments and social responsibility to consumers. By adopting eco-friendly materials, green production processes, and third-party certifications, brands can send signals to consumers about their dedication to sustainable development. These signals can enhance consumer trust and goodwill, which ultimately influence purchasing decisions.

However, there are still shortcomings in understanding the diversity of eco-positioning strategies, consumer psychological mechanisms, differences among consumer groups, long-term effects, and cultural adaptability. The Signaling Theory, TPB, and ELM are integrated with eco-positioning strategies. This study explores how eco-positioning influences consumer attitudes, behavioral intentions, and trust signals across different consumer groups, ultimately affecting their purchasing behaviors. These theories offer a solid theoretical foundation for understanding the practical effects of eco-positioning in fashion marketing.

## Research methodology

### Fashion marketing and consumer-related theories under Eco-positioning

Fashion marketing refers to the process by which enterprises promote fashion products or brands to consumers through various marketing strategies and tactics. It encompasses product design, brand positioning, advertising, market promotion, sales channel management, and other aspects. Fashion marketing focuses on the aesthetics and functionality of products. Meanwhile, it involves shaping brand image, understanding market demand, and analyzing consumer psychology. Effective fashion marketing strategies can help brands stand out in competitive markets, attract target consumers, enhance brand awareness, and increase market share^51^.

**Fig. 1:**
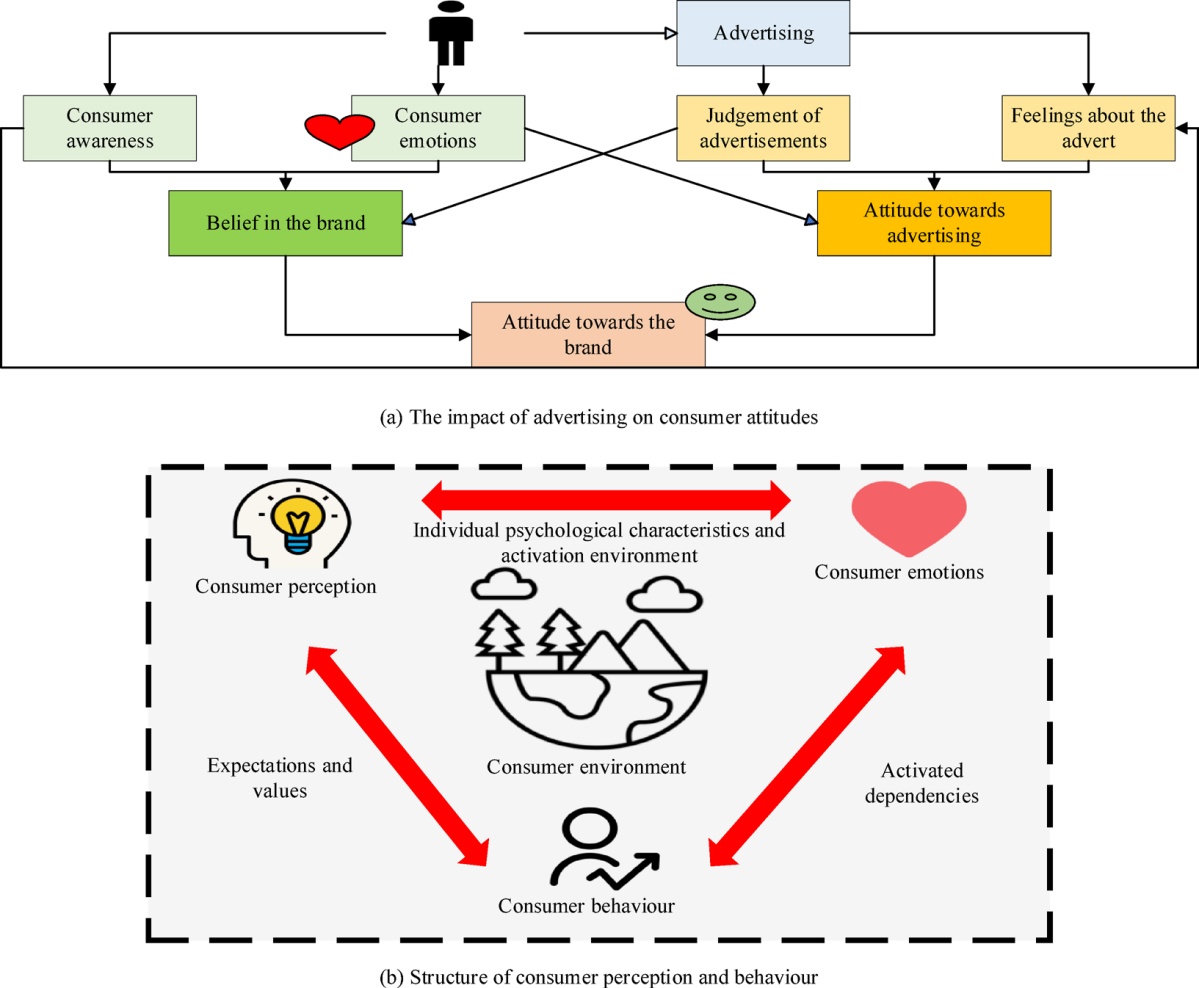
The influence of advertising on consumer attitudes and the structure of consumer perception and behavior.

Consumer perception refers to the beliefs, attitudes, and behavioral patterns that consumers form during encountering, evaluating, and purchasing products or services. It encompasses consumer awareness of brands, evaluations of product quality, understanding of market trends, and responses to advertising and promotional activities. Consumer perception is influenced by factors such as personal experience, social influences, and cultural background. Understanding perception cognition can assist enterprises in devising more precise marketing strategies, enhancing brand image, and driving sales growth. By studying consumer perception, enterprises can better meet consumer needs, optimize products and services, and improve market competitiveness^52^. Consumer behavior is the various behaviors and decision-making processes that they exhibit when selecting, purchasing, using, and evaluating products or services. It includes consumer needs recognition before purchasing, information search, evaluation of options, purchase decision-making, and post-purchase usage experience and feedback^53^. Consumer willingness refers to the price consumers are willing to pay for a product or service, the degree to which they make a purchase, or their attitude toward participating in specific consumption activities. These factors are critical when making purchasing decisions. Consumer willingness reflects consumers’ recognition of the value of products and their expectations regarding price, brand, quality, and other factors^54^. The impact of advertising on consumer attitudes and the structure of consumer perception and behavior are illustrated in Fig. 1^55,56^.

Eco-positioning is a brand strategy in marketing where brands establish an eco-friendly image in the market through measures such as using eco-friendly materials, green production processes, and social responsibility initiatives. Sustainable fashion encompasses comprehensive environmental protection and social fairness practices throughout the entire lifecycle of fashion products, from design to disposal. As a component of the brand strategy, eco-positioning helps shape the brand’s environmental image and attracts environmentally conscious consumers. Sustainable fashion represents a broader concept encompassing various environmental and social responsibility initiatives within the industry. Together, eco-positioning enhances the brand’s environmental image among consumers, while sustainable fashion promotes comprehensive environmental and social responsibility practices across the industry. These two elements collectively drive the fashion industry toward greater sustainability^57^.

A Likert scale is a commonly used questionnaire tool to measure the intensity of respondents’ attitudes or opinions towards a statement. The Likert scale quantifies respondents’ opinions into data by setting multiple levels from “strongly disagree” to “strongly agree”, facilitating analysis and comparison. Typically consisting of 5 or 7 levels, each corresponds to a score reflecting the degree of respondents’ attitudes towards the statement. This tool is simple and user-friendly while helping researchers gain insights into trends and preferences in respondents’ attitudes. Thus, this tool is widely applied in market research, psychological surveys, and social science research^58–60^.

### Research design on the impact of Eco-positioning strategies on consumers

This study adopts a modular multi-experiment design using a shared participant pool. Each participant completed three distinct between-subject experimental modules in randomized order, focusing respectively on: (1) brand evaluation, (2) willingness to pay, and (3) sustainable fashion consumption intention.

In Module 1, participants were randomly assigned to one of three advertising conditions: product-related eco-positioning, process-related eco-positioning, or no advertisement (control). Following exposure to the assigned stimulus, they evaluated the brand and indicated their purchase intention. Brand familiarity was measured as a potential moderator. In Module 2, participants were randomly assigned to one of three product description conditions representing varying levels of eco-positioning intensity: no eco-related content, moderate eco-positioning (featuring basic eco-friendly attributes), and strong eco-positioning (featuring comprehensive sustainability claims). Participants then reported their willingness to pay and perceived environmental sustainability of the described products. In Module 3, participants were randomly assigned to view one of three informational stimuli: an eco-themed fashion advertisement highlighting sustainability commitments, a neutral fashion-related message without ecological emphasis, or no message (control). They subsequently responded to items assessing sustainable fashion consumption intention, while environmental concern and fashion involvement were measured as potential moderating variables. 

 Across all modules, participants were independently randomised to experimental conditions, and each module employed distinct manipulation materials and outcome measures to prevent carryover effects. 

## Experimental design and performance evaluation

### Dataset collection and experimental environment

This study employs an online experiment to investigate the impact of eco-positioning strategies on consumer behavior. For participant recruitment, the research team collected data through the SoJump platform. To ensure sample representativeness and experimental validity, random sampling was used to recruit participants from the platform’s user pool. Additionally, the following screening criteria were established to enhance data quality and ensure the accuracy of experimental results. Participants must be at least 18 years old to ensure they possess legal purchasing decision-making capabilities. Participants must be active fashion consumers, having purchased fashion products at least once in the past three months. To effectively test the influence of eco-positioning strategies, participants were required to answer questions about environmental awareness during the preliminary screening phase. Meanwhile, only those with high environmental concern were allowed to proceed with the experiment. During the recruitment process, 123 participants were recruited through platform advertisements and social media promotions, with 102 valid questionnaires ultimately collected, yielding a response rate of 82.9%.

The sample size is determined based on multiple factors. First, referencing prior studies in similar fields, sample sizes between 100 and 150 are common in comparable online experiments to ensure sufficient statistical power for data analysis. According to G*Power analysis, this study assumes the use of regression analysis with an effect size of 0.15 (medium effect), a significance level of 0.05, and a statistical power of 0.80. Based on these parameters, the minimum required sample size is 89. Therefore, the sample size of 102 is sufficient to meet statistical analysis requirements and demonstrates high reliability and representativeness. Furthermore, the sample is drawn from the SoJump platform, where users exhibit diverse socioeconomic backgrounds and consumption behaviors, covering various age groups, professions, and consumption levels. This ensures sample diversity and the generalizability of the research findings. Thus, the 102 valid questionnaires are deemed adequate and reasonable.

In this study, a questionnaire is used to collect relevant data from participants. The questionnaire items are divided into three main sections: brand evaluation, willingness to pay, and sustainable consumption intention. To ensure the validity and reliability of the questionnaire, some items are adapted from previous studies; Others are specifically designed based on the objectives of this study. The brand evaluation section utilized scales from Park et al. (2013) to measure brand attitude and trust^61^. The willingness-to-pay section referenced scales from Biswas et al. (2016) on willingness to pay for green products^62^. The sustainable consumption intention section is newly designed based on the specific objectives of this study, developed through discussions with environmental experts. All borrowed scales are appropriately modified to align with this study’s fashion context and objectives. The complete version is provided in the appendix to facilitate readers’ evaluation of the questionnaire’s structure and content. It includes detailed descriptions of each question, the corresponding scale type (Likert scale), and the scoring method for each item.

During the experiment, participants were first asked to complete a questionnaire on their personal information, environmental awareness, and fashion consumption behaviors. They then completed three experimental modules presented in randomized order. Each module included exposure to stimulus materials specific to its manipulation condition, followed by outcome measures relevant to the module. Their responses were based on the content of the stimuli and their perceptions of the brand, product, or sustainability message. The questionnaire utilized a Likert scale to measure consumers’ attitudes and behavioral intentions towards eco-positioning. All experimental data were collected in real-time through the SoJump platform and analyzed using SPSS software. In addition, the personal information of all participants is strictly confidential. The study has been approved by an ethics review committee to ensure compliance with relevant ethical and privacy regulations. The reliability coefficient is 0.81, and the validity is 0.84, indicating the data’s reliability and validity.

The reliability coefficient of 0.81 mentioned here refers to Cronbach’s alpha coefficient of the scale, which is used to assess the internal consistency of the questionnaire. Cronbach’s alpha value is commonly employed to measure the consistency and reliability among items within a scale. The value typically ranges from 0 to 1, with higher values indicating stronger internal consistency of the scale. In this study, Cronbach’s alpha value of 0.81 demonstrates that the scale used exhibits good internal consistency. To evaluate the scale validity, this study employs tests for construct validity and Average Variance Extracted (AVE). Construct validity assesses whether the scale accurately measures the theoretical construct it intends to measure. AVE evaluates the degree of association between individual items and the latent variable, with higher AVE values indicating stronger convergent validity. For discriminant validity, this study ensures the scale’s good discriminant validity by assessing the correlations between latent variables and comparing factor loadings and cross-loadings. Moreover, Confirmatory Factor Analysis (CFA) validates the scale’s validity. In the CFA, all factor loadings exceed 0.6, indicating good construct validity of the scale.

**Fig. 2:**
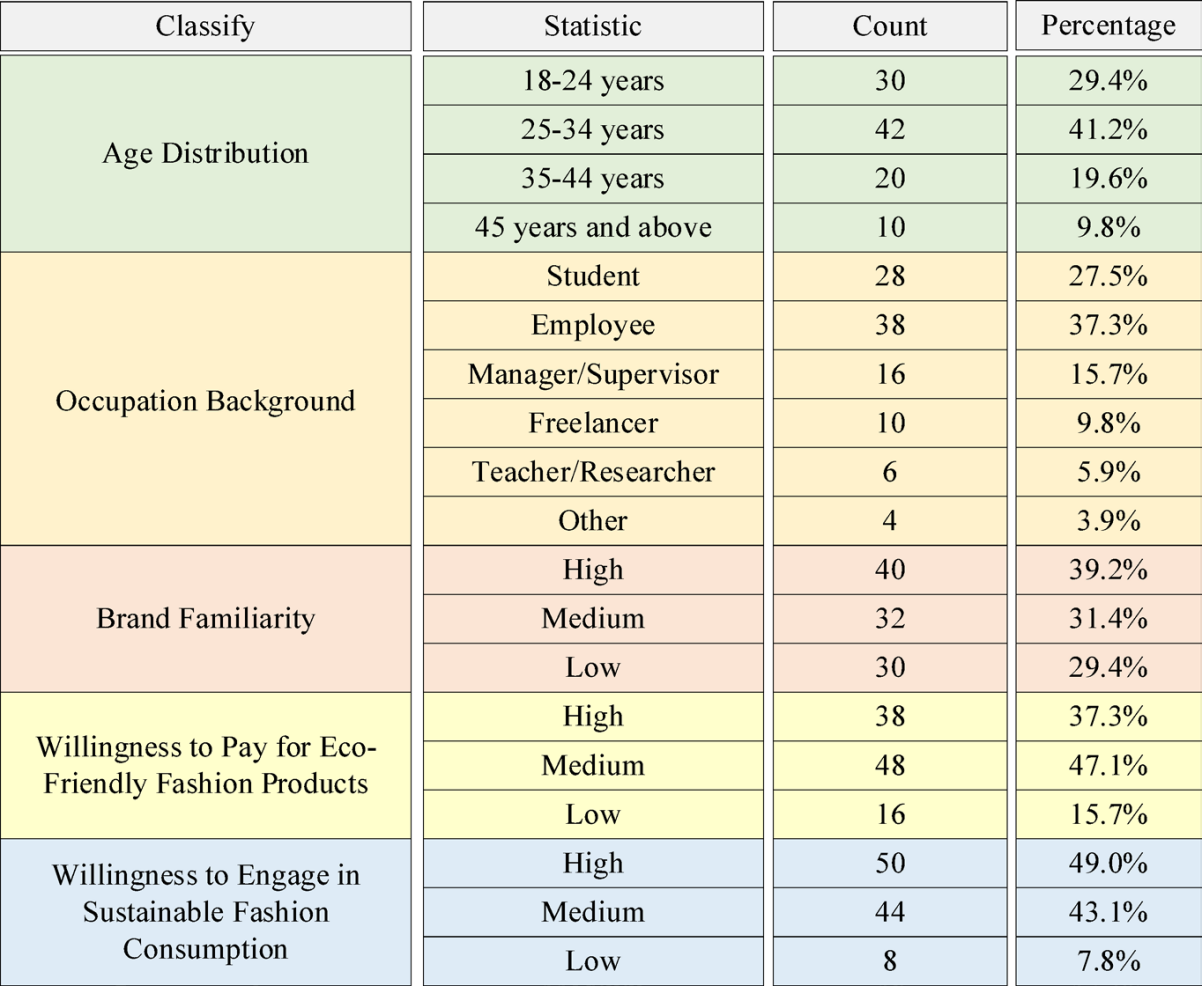
Descriptive statistical results of the questionnaire.

The questionnaire’s descriptive statistical results are depicted in Fig. 2.

The hardware and software environments include Windows 10 operating on a 64-bit system with 8GB RAM and an Intel i7-7500 Central Processing Unit (CPU). Software-wise, the SoJump platform is used for questionnaire design and data collection. Meanwhile, Statistical Product and Service Solutions (SPSS) 26.0 software is employed for reliability and validity testing and descriptive statistical analysis.

### Performance evaluation

The data were analyzed across three independent experimental modules. Each module’s manipulation and dependent variables were evaluated separately.

**Fig. 3:**
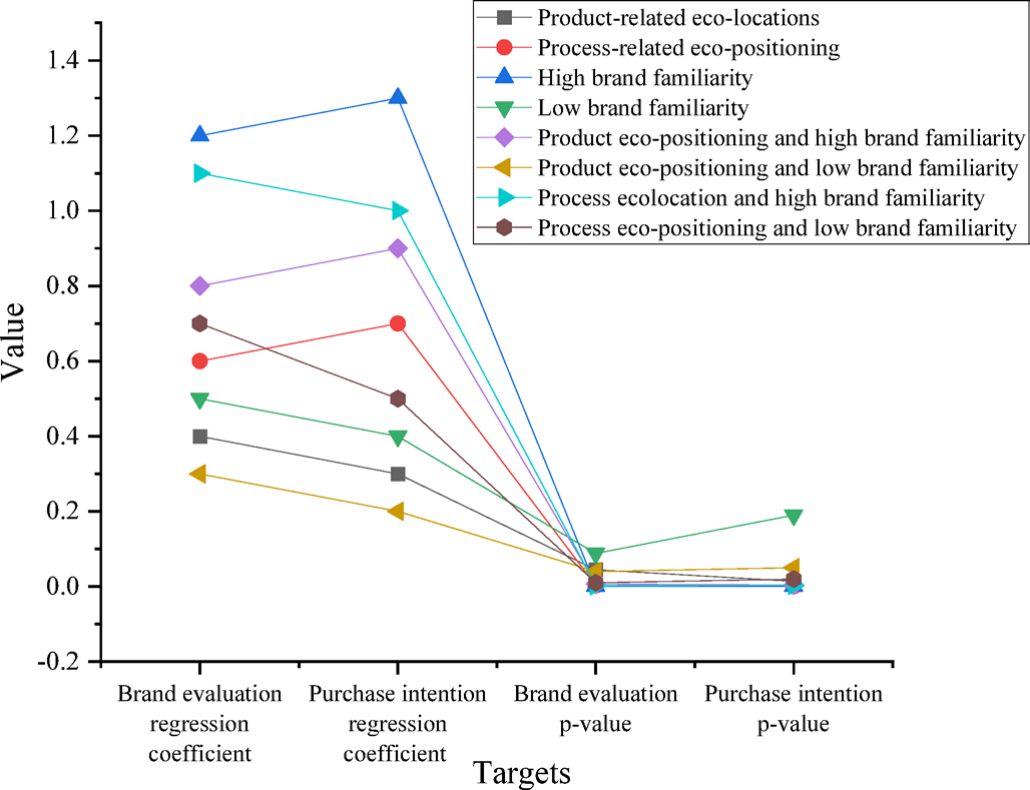
The influence of eco-positioning strategy on brand evaluation results.

The influence of eco-positioning strategies on brand evaluation is shown in Fig. 3. The study results indicate that product-related and process-related eco-positioning substantially influence brand evaluation and purchase intentions. Specifically, for product-related eco-positioning, the regression coefficients for brand evaluation and purchase intentions are 0.4 and 0.3, with p-values of 0.045 and 0.014. For process-related eco-positioning, the regression coefficient for brand evaluation is 0.6 (p-value = 0.003), and for purchase intentions, it is 0.7 (p-value = 0.001). Brand familiarity also plays a significant moderating role, where under high brand familiarity, the regression coefficients for brand evaluation and purchase intentions are 1.2 and 1.3, and the p-values are all 0.001. However, under low brand familiarity, the regression coefficients for brand evaluation and purchase intentions reach 0.5 and 0.4, and their p-values are 0.088 and 0.19. When combined with brand familiarity, the regression coefficient of brand evaluation for product eco-positioning and high brand familiarity is 0.8, with a p-value of 0.007, and the purchase intention’s regression coefficient is 0.9, with a p-value of 0.004; The regression coefficient is 1.1 (p-value = 0.002) for process eco-positioning and high brand familiarity, for purchase intention is 1.0, (p-value = 0.003). This indicates that process-related eco-positioning has a more prominent impact on brand evaluation and purchase intentions, while high brand familiarity enhances the positive effects of eco-positioning strategies.

The regression analysis results are presented in Table 2. The regression coefficients, effect sizes, and related confidence intervals (CI) and VIF for multiple influencing factors are considered in the regression analysis. First, in terms of regression coefficients, both product-related and process-related eco-positioning significantly impact brand evaluation and purchase intention, with strong effects. Their Cohen’s f² values are 0.15 and 0.25, respectively, indicating moderate effect sizes in the model. The regression coefficient for high brand familiarity is 1.2, with an effect size of 0.55, demonstrating a significant and strong influence on purchase intention and brand evaluation. In contrast, the impact of low brand familiarity is smaller, with a regression coefficient of 0.5 and an effect size of only 0.10. This indicates that eco-positioning has a diminished effect on consumer decision-making when brand familiarity is low. Regarding CIs, none of the regression coefficients’ CIs cross zero, further validating their significance and enhancing the credibility of the results. For example, the CI for process-related eco-positioning is [0.28, 0.92], demonstrating a significant and stable positive impact on consumer purchase intention. In terms of effect sizes (Cohen’s f²), all models exhibit moderate to strong effects. For instance, the effect size for high brand familiarity is 0.55, significantly exceeding the threshold of 0.35, highlighting its substantial role in driving consumer purchase intention. In contrast, the effect size for low brand familiarity is only 0.10, reflecting its weaker influence. Multicollinearity analysis reveals that all variables’ VIF values are below 5 (specifically ranging from 2.1 to 2.5), indicating no severe multicollinearity issues in the model. Therefore, the proposed regression model is statistically stable and reliable.

**Table 2:** Results of regression analysis.

Variable	regression coefficient	*p*-value	CI (95%)	Cohen’s f²	VIF
Product-related eco-positioning	0.4	0.045	[0.02, 0.78]	0.15	2.1
Process-related eco-positioning	0.6	0.003	[0.28, 0.92]	0.25	2.3
High brand familiarity	1.2	0.001	[0.88, 1.52]	0.55	1.9
Low brand familiarity	0.5	0.088	[−0.02, 1.02]	0.10	2.4
Product eco-positioning and high brand familiarity	0.8	0.007	[0.25, 1.35]	0.32	2.0
Product eco-positioning and low brand familiarity	0.3	0.04	[0.05, 0.55]	0.08	2.5
Process eco-positioning and high brand familiarity	1.1	0.002	[0.56, 1.64]	0.38	2.2
Process eco-positioning and low brand familiarity	0.7	0.01	[0.23, 1.17]	0.22	2.3

**Fig. 4:**
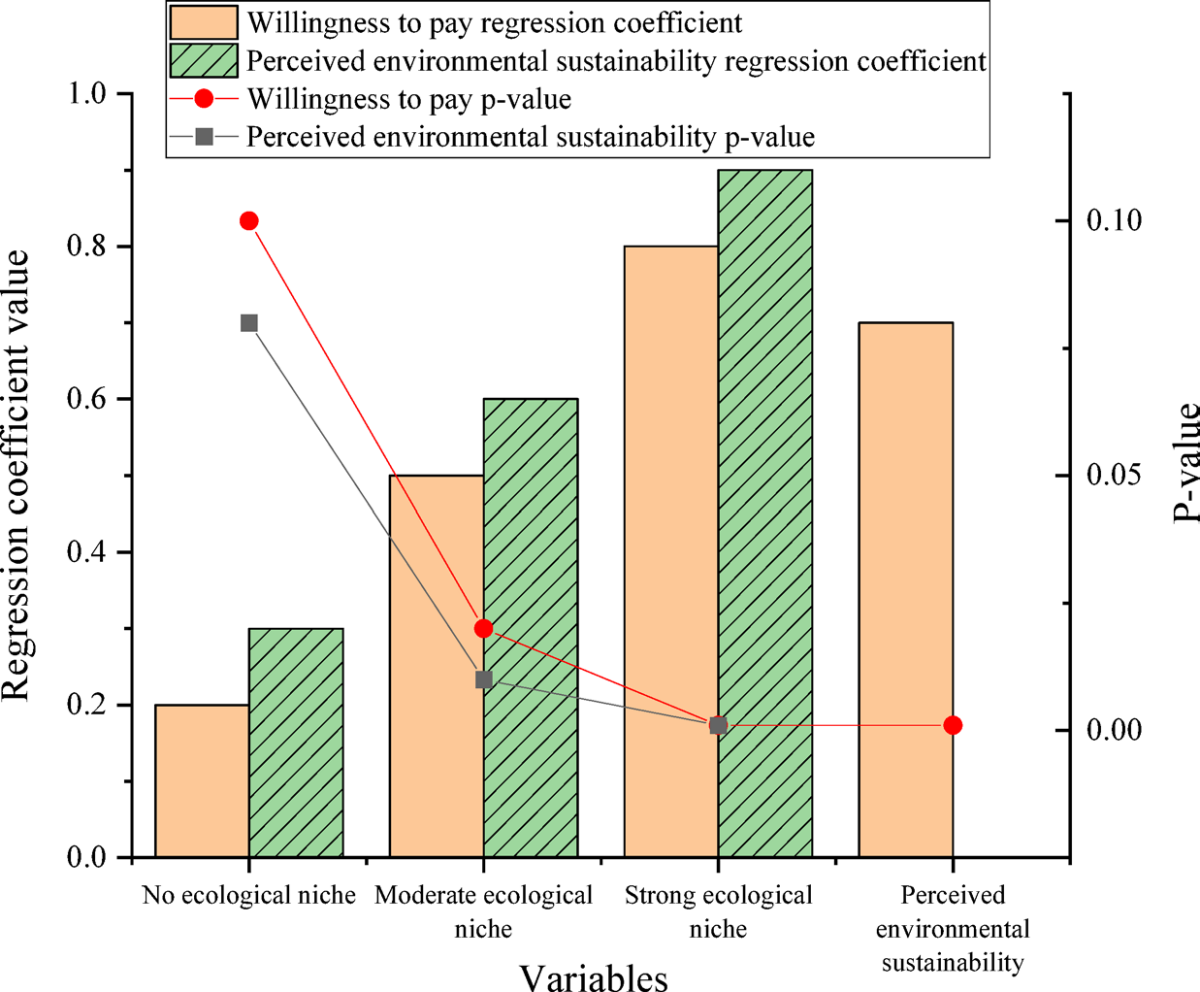
The impact of eco-positioning on willingness to pay.

In Module 2 (willingness to pay), participants were exposed to product descriptions containing varying levels of eco-positioning: no eco-related content, moderate eco-positioning (e.g., simple eco-friendly features), and strong eco-positioning (e.g., comprehensive sustainability claims). The dependent variables included willingness to pay and perceived environmental sustainability. The impact of eco-positioning on willingness to pay is suggested in Fig. 4.

Figure 4 shows that under no eco-positioning conditions, the regression coefficients for willingness to pay and perceived environmental sustainability are 0.2 and 0.3 (p-values = 0.1 and 0.08). Under moderate eco-positioning conditions, the regression coefficients for both variables are 0.5 and 0.6 (p-values = 0.02 and 0.01). Under strong eco-positioning conditions, the two variables’ regression coefficients are 0.8 and 0.9, with p-values of 0.001 and 0.001. Perceived environmental sustainability remarkably mediates the effect of eco-positioning on willingness to pay, with a regression coefficient of 0.7 (p-value = 0.001). The study demonstrates that eco-positioning strategies effectively enhance consumer willingness to pay by increasing perceived environmental sustainability.

**Fig. 5:**
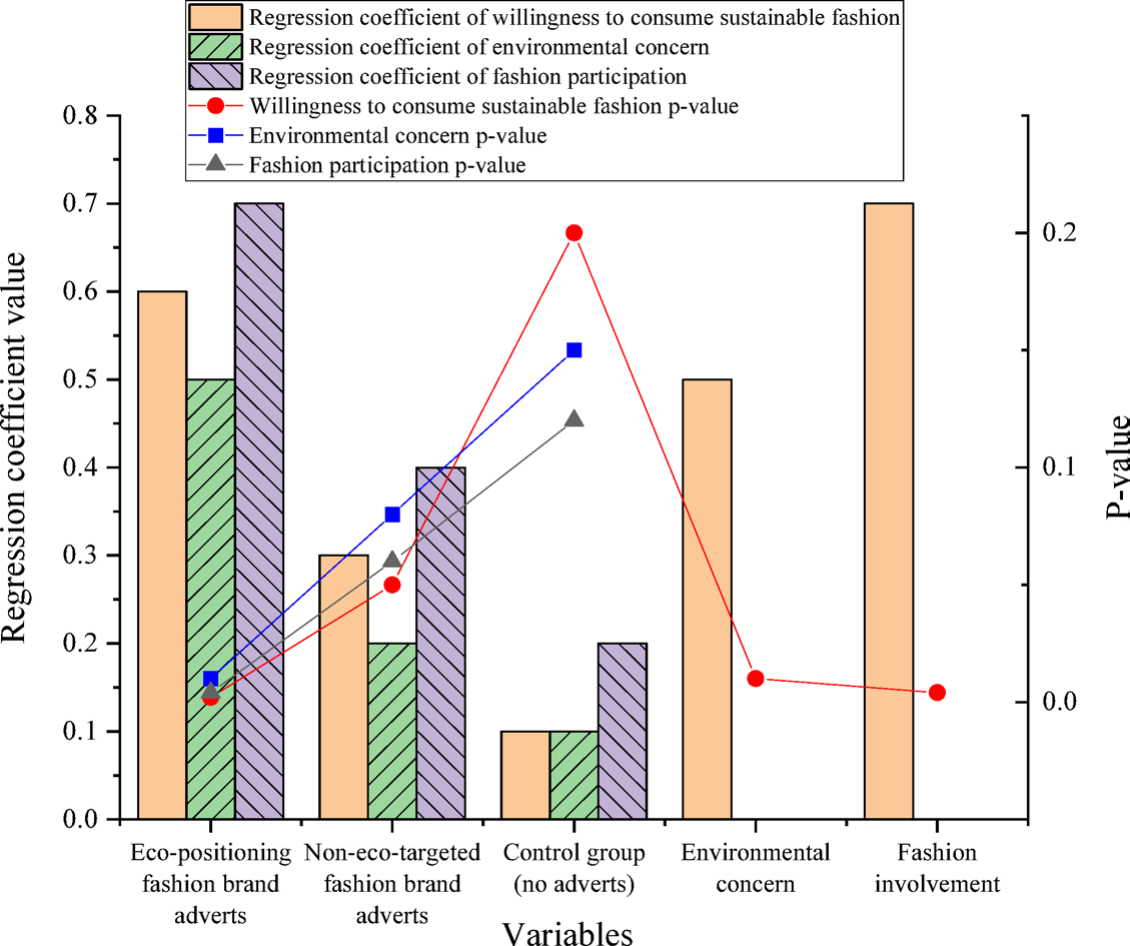
The effect of eco-positioning on sustainable fashion consumption intention.

In Module 3 (sustainable consumption intention), participants viewed either an eco-themed fashion advertisement highlighting sustainability commitments, a neutral fashion-related message without ecological emphasis, or no message (control). The dependent variables included sustainable fashion consumption intention, with environmental concern and fashion involvement measured as potential moderators. The eco-positioning’s effect on sustainable fashion consumption intention is denoted in Fig. 5.

Figure 5 reveals that under eco-positioning fashion brand advertisement conditions, the regression coefficients for sustainable fashion consumption intention, environmental concern, and fashion involvement reach 0.6, 0.5, and 0.7; Their corresponding p-values are 0.002, 0.01, and 0.004, respectively. Under non-eco-positioning conditions, the regression coefficients for the above three variables are 0.3, 0.2, and 0.4 (p-values = 0.05, 0.08, and 0.06). In the control group (no advertisement), the three variables’ regression coefficients are 0.1, 0.1, and 0.2, with p-values of 0.2, 0.15, and 0.12. Moreover, environmental concern and fashion involvement markedly moderate the relationship between eco-positioning and sustainable fashion consumption intention. The regression coefficients are 0.5 (p-value = 0.01) for environmental concern and 0.7 (p-value = 0.004) for fashion involvement. These findings illustrate that eco-positioning strategies significantly promote consumers’ sustainable fashion consumption intention, with environmental concern and fashion involvement playing crucial moderating roles.

### Discussion

The main findings of this study indicate that eco-positioning strategies have a significant impact on consumers’ brand evaluation, willingness to pay, and sustainable consumption intentions. Among these strategies, process-oriented eco-positioning has a more pronounced effect on enhancing brand evaluation and purchase intention, while brand familiarity plays a crucial moderating role in this process. These findings should be reassessed within the context of existing literature to highlight their theoretical contributions. First, the findings regarding differences in eco-positioning types engage in a dialogue with the study by Majeed et al. (2022)^63^. Although their research suggested that eco-positioning enhanced purchase intention by improving a brand’s environmental image, it did not differentiate between product-oriented and process-oriented strategies. Through experimental validation, this study demonstrates their distinct psychological effects on consumers, particularly revealing the advantages of process-oriented strategies (e.g., green production processes and third-party certifications) in shaping brand credibility and perceived commitment. This conclusion aligns with the findings of Shanti et al. (2022) on the impact of sustainable practices in the hotel industry on brand equity^64^. Their study similarly found that consumers’ attention to a company’s full-process environmental responsibility significantly influenced brand perception, indicating cross-industry commonalities. Second, this study’s analysis of the moderating role of brand familiarity further extends the theoretical framework of Kassie et al. (2023). While their research emphasized the fundamental role of brand familiarity in green marketing^65^, it did not explore the dynamic moderating mechanisms of familiarity under different eco-positioning strategies. This study indicates that within process-oriented strategies, high brand familiarity significantly amplifies consumer trust in environmental commitments, whereas low-familiarity brands rely more on specific certification signals (e.g., third-party endorsements). This finding is consistent with signal theory, which posits that the credibility of information interacts with the recipient’s prior knowledge to influence perception. Furthermore, the findings of this study complement the TPB by demonstrating that process-oriented strategies enhance consumers’ perceived control over brand environmental actions (e.g., transparent production processes), thereby indirectly strengthening subjective norms and perceived behavioral control. This mechanism explains why process-oriented strategies have a stronger effect compared to product-oriented strategies. Notably, previous studies have identified the direct impact of eco-labels on willingness to pay. However, this study employs SEM to reveal the mediating role of perceived environmental sustainability, providing a more granular explanation for the application of signal theory in fashion marketing. Overall, by integrating multiple theoretical perspectives with experimental design, this study validates key arguments in the existing literature (e.g., the relationship between brand familiarity and environmental commitments). Meanwhile, it addresses critical gaps in the typological comparison of eco-positioning strategies, dynamic moderating effects, and psychological mechanism pathways. These contributions establish a scalable analytical framework for future research.

This study also has certain limitations. While it identifies the significant impact of eco-positioning on consumer behavior, the sample composition and experimental design present constraints on generalizability. The current experimental parameters preclude thorough exploration of potential moderating factors beyond those explicitly measured, such as consumers’ cultural backgrounds, socioeconomic status, and prior environmental knowledge. Additionally, the controlled laboratory-like environment of online experiments may not fully capture the complexity of real-world purchasing contexts where multiple competing factors simultaneously influence consumer decisions. Future research could validate these findings in broader samples, especially across diverse cultural contexts, where consumer responses to eco-positioning strategies may vary. Theoretically, this study provides a multidimensional understanding of how eco-positioning strategies influence consumer decision-making processes by integrating TPB, ELM, and Signaling Theory. TPB explains how consumers form purchase intentions through attitudes, subjective norms, and perceived behavioral control when encountering eco-positioning. ELM helps understand how consumers may process ecological information through central or peripheral routes depending on the level of information provided. Signaling Theory offers a framework for how brands convey environmental signals through eco-positioning. By combining these theories, this study enriches the theoretical foundation of eco-marketing. Meanwhile, it provides more systematic theoretical guidance for fashion brands in developing sustainable marketing strategies.

This study delves into the impact of eco-positioning in the fashion marketing context on consumer perceptions and behaviors. It enriches the theoretical literature on sustainable fashion marketing, green advertising, and consumer behavior. It also enhances the theoretical understanding of how different eco-positioning strategies shape consumer responses and how brand familiarity moderates these effects. The study reveals that perceived environmental sustainability mediates the relationship between eco-positioning and consumer willingness to pay. This finding offers new perspectives on the application of Signaling Theory, ELM, Schema Theory, and TPB in sustainable fashion marketing. In practical management, the study provides valuable recommendations for fashion brands to develop effective eco-positioning strategies to communicate environmental commitments, enhance brand awareness, and promote sustainable consumption. It emphasizes the need for strategies to fully consider brand familiarity and consumer characteristics, such as environmental awareness and fashion involvement, to maximize market effectiveness.

## Conclusions

### Research contribution

#### Theoretical contribution

This study enriches the theoretical foundation of sustainability and eco-positioning in the field of fashion marketing. First, it provides a multidimensional theoretical perspective on how consumers make decisions through eco-positioning by integrating TPB, ELM, and Signaling Theory. In particular, this study highlights the importance of process-related eco-positioning in brand evaluation and purchase intention. This result deepens the understanding of consumers’ environmental attitudes toward fashion brands. Second, this study offers a new perspective for the theoretical development of eco-positioning by proposing the moderating role of brand familiarity in eco-marketing strategies. This finding provides new theoretical hypotheses for future research.

This study adopts a mixed research method of a multi-module experimental design and questionnaire surveys, effectively isolating and examining the impact of eco-positioning strategies on consumer behavior. By systematically analyzing different eco-positioning strategies (product-related and process-related) and brand familiarity, this study provides an actionable empirical research framework for subsequent research. Especially in the fashion industry, this framework can help researchers design more accurate consumer behavior studies. The impact path of eco-positioning on consumer willingness to pay is analyzed through SEM, providing empirical evidence for the application of such statistical methods in fashion marketing.

#### Practical contribution

This study provides practical guidance for fashion brands implementing sustainable marketing strategies. First, the findings indicate that process-related eco-positioning remarkably influences brand evaluation and purchase intention. This suggests that brand managers can enhance consumers’ brand identification by emphasizing environmental measures in the production process. Second, brand familiarity plays a significant moderating role in the effectiveness of eco-positioning. This implies that brands must adjust their strategies based on their market awareness and recognition when formulating eco-marketing strategies. Finally, the study also shows that eco-positioning increases consumers’ willingness to pay and effectively enhances their intention to engage in sustainable fashion consumption. Therefore, brands can attract environmentally conscious consumer groups and promote green consumption behaviors through more explicit eco-positioning strategies.

### Future works and research limitations

A limitation of this study is its focus on the short-term impacts of eco-positioning on consumers’ immediate perceptions and behavioral intentions. It does not fully explore the long-term effects of eco-positioning on fashion brand loyalty and actual purchase behavior. Future research could explore the long-term impacts of eco-positioning on fashion industry brand loyalty and actual purchase behavior. Additionally, investigating the roles of cultural factors and individual differences in shaping consumer responses to eco-positioning would provide a more nuanced understanding of sustainable fashion consumption.

## Appendix

Questionnaire:

Thank you for participating in this study. This questionnaire aims to understand consumers’ attitudes towards eco-friendly fashion products, purchase intention, and sustainable fashion consumption behavior. This questionnaire is divided into five parts, all questions are multiple choice. Your answers will play an important role in this study. Please fill in according to the actual situation. Your answers will be strictly confidential and only be used for academic research.

### Part 1: Basic personal information

1. **Your age range**:

- (A) 18–24 years old.
- (B) 25–34 years old.
- (C) 35–44 years old.
- (D) 45 years old and above.

2. **Your professional background**:

- (A) Students.
- (B) Employees (including full-time and part-time).
- (C) Manager/Supervisor.
- (D) Freelancers.
- (E) Teachers/Researchers.
- (F) Other (please specify: ________).

### Part 2: Brand familiarity

3. **Your familiarity with the following fashion brands is**:

- (A) Highly familiarity.
- (B) Medium familiarity.
- (C) Low familiarity.
- (D) Completely unfamiliar.

### Part 3: Willingness to pay for eco-friendly fashion products

4. **Are you willing to pay more for eco-friendly fashion products?**

- (A) Very willing.
- (B) More willing.
- (C) Not very willing.
- (D) Completely unwilling.

5. **If you are willing to pay more for eco-friendly fashion products**,** please choose an additional payment amount that you can accept**:

- (A) Not exceeding 10%.
- (B) 10–20%.
- (C) 20–30%.
- (D) More than 30%.

### Part 4: Sustainable fashion consumption intention

6. **Are you willing to participate in sustainable fashion consumption (such as purchasing eco-friendly**,** recyclable fashion products**,** or choosing products that the brand promises sustainable development)?**

- (A) Very willing.
- (B) More willing.
- (C) Not very willing.
- (D) Completely unwilling.

7. **Are you willing to adjust your shopping habits (such as choosing eco-friendly brands**,** participating in recycling**,** etc.) to purchase sustainable fashion products?**

- (A) Very willing.
- (B) More willing.
- (C) Not very willing.
- (D) Completely unwilling.

### Part 5: Eco-positioning advertising evaluation

Please evaluate your opinion of the relevant advertisement based on the description below. The following questions are relevant to the brand’s eco-positioning advertising, and your answers will help understand consumer attitudes.

8. **What is your attitude toward the environmental behaviors displayed by the brand in advertising**,** such as the use of eco-friendly materials**,** green production processes**,** and social responsibility?**

- (A) Very positive.
- (B) More positive.
- (C) Neutral.
- (D) Relatively negative.
- (E) Very negative.

9. **How much do you agree with the brand’s ecological positioning strategy**,** such as eco-friendly production processes or the use of eco-friendly materials?**

- (A) Strongly agree.
- (B) Generally agree.
- (C) Neutral.
- (D) Generally disagree.
- (E) Strongly disagree.

10. **After seeing advertisements for eco-friendly fashion brands**,** would you be more willing to purchase their products?**

- (A) Very willing.
- (B) More willing.
- (C) Not very willing.
- (D) Completely unwilling.

### Part 6: Consumer environmental concern and fashion participation

Please choose the option that best suits your situation based on your environmental concern and fashion participation.

11. **How concerned are you about environmental issues?**

- (A) Very concerned.
- (B) More concerned.
- (C) General attention.
- (D) Rarely concerned.
- (E) Not paying attention at all.

12. **How concerned are you about fashion products?**

- (A) Very concerned.
- (B) More concerned.
- (C) General attention.
- (D) Rarely concerned.
- (E) Not paying attention at all.

### Part 7: Additional questions

13. **How do you view the future development of eco-friendly fashion products?**

- (A) Very optimistic.
- (B) More optimistic.
- (C) Neutral.
- (D) Not very optimistic.
- (E) Very pessimistic.

14. **How do you think brands should improve their environmental marketing strategies?**

- (A) Improve environmental transparency.
- (B) Increase environmental certification or third-party certification.
- (C) Strengthen the eco-friendly production process.
- (D) Better educate consumers to understand eco-friendly fashion.
- (E) Other (please specify: ________).

Thank you for your participation!

## Data Availability

The datasets used and/or analyzed during the current study are available from the corresponding author Ziqi Zhong on reasonable request via e-mail Z.Zhong6@lse.ac.uk.
